# Effects of habitat types on the dynamic changes in allocation in carbon and nitrogen storage of vegetation–soil system in sandy grasslands: How habitat types affect C and N allocation?

**DOI:** 10.1002/ece3.7751

**Published:** 2021-06-05

**Authors:** Peng Lv, Shanshan Sun, Eduardo Medina‐Roldánd, Shenglong Zhao, Ya Hu, Aixia Guo, Xiaoan Zuo

**Affiliations:** ^1^ Naiman Desertification Research Station Northwest Institute of Eco‐Environment and Resources Chinese Academy of Sciences Lanzhou China; ^2^ Urat Desert‐grassland Research Station Northwest Institute of Eco‐Environment and Resources Chinese Academy of Sciences Lanzhou China; ^3^ University of Chinese Academy of Sciences Beijing China; ^4^ Department of Health and Environmental Sciences Xi'an Jiao Tong‐Liverpool University Suzhou China

**Keywords:** C and N storage, habitat types, sandy grassland, vegetation restoration, vegetation–soil system

## Abstract

The progressively restoration of degraded vegetation in semiarid and arid desertified areas undoubtedly formed different habitat types. The most plants regulate their growth by fixing carbon with their energy deriving from photosynthesis; carbon (C) and nitrogen (N) play the crucial role in regulating plant growth, community structure, and function in the vegetation restoration progress. However, it is still unclear how habitat types affect the dynamic changes in allocation in C and N storage of vegetation–soil system in sandy grasslands. Here, we investigated plant community characteristics and soil properties across three successional stages of habitat types: semi‐fixed dunes (SFD), fixed dunes (FD), and grasslands (G) in 2011, 2013, and 2015. We also examined the C and N concentrations of vegetation–soil system and estimated their C and N storage. The C and N storage of vegetation system, soil, and vegetation–soil system remarkably increased from SFD to G. The litter C and N storage in SFD, N storage of vegetation system in SFD, and N storage of soil and vegetation–soil system in FD increased from 2011 to 2015, while aboveground plant C and N storage of FD were higher in 2011 than in 2013 and 2015. Most of C and N were sequestered in soil in the vegetation restoration progress. These results suggest that the dynamic changes in allocation in C and N storage in vegetation–soil systems varied with habitat types. Our study highlights that SFD has higher N sequestration rate in vegetation, while FD has the considerably N sequestration rate in the soil.

## Graphical abstract

It is still unclear how habitat types affect the dynamic changes in allocation in C and N storage of vegetation–soil system in sandy grasslands. Here, we investigated plant community characteristics and soil properties across three successional stages of habitat types: semi‐fixed dunes (SFD), fixed dunes (FD), and grasslands (G) in 2011, 2013, and 2015. Our results suggest that the dynamic changes in allocation in C and N storage in vegetation–soil systems varied with habitat types. Our study highlights that SFD has higher N sequestration potential in vegetation, while FD has the considerably N sequestration potential in the soil.

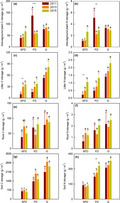

## 1 INTRODUCTION

As one of the largest vegetation types in terrestrial ecosystem, grassland occupies about 40% of earth's land surface (Kemp et al., 2013; Liu & Zhang, 2018). Grassland has a great effect maintaining the function of terrestrial ecosystem, such as biodiversity, livestock productivity, and ecosystem services (Chen et al., 2017; Ding et al., 2017; Fu et al., 2018). However, the anthropogenic disturbances cause the changes in plant community structure and function, then further leading to extensive degradation of grassland (Guo et al., 2019). Livestock grazing is the most widely measure or management practice for grassland utilization, which strongly affects plant community structure and function (Bai et al., 2012; He et al., 2020; Yang et al., 2013; Zhou et al., 2017). Long‐term grazing or overgrazing can decrease the plant biomass and the cycling of carbon (C) and nitrogen (N) in grassland ecosystems (He et al., 2020; Moinardeau et al., 2019; Wu et al., 2012; Yang et al., 2019), which is regarded as the main reason for the extensive degradation of grassland in drylands (Bai et al., 2012). Thus, to reduce grazing pressure or exclude grazing disturbance is crucial to restore the degraded grassland and the sustainable management of grassland ecosystem.

The enclosure or livestock exclusion has become an important or effective measure, which has been widely used for the restoration of degraded grassland ecosystems (Deng et al., 2017; Zhao et al., 2019). As a result, habitat conversions were occurred in the restoration progress of degraded grassland. The C and N are essential elements for the development of all organisms on earth, which are important to regulate the terrestrial ecosystems' structure and function (Elser et al., 2010; Wang et al., 2016). Some studies have shown that the enclosure could increase the soil organic C and available N, the biomass and total soil C and N with light fraction C and N responding more rapidly than total soil C and N to grazing exclusion and vegetation recovering faster than soil (Abdalla et al., 2018; Chen et al., 2012; Hu et al., 2016), while other studies have demonstrated that there is negative or no effects of grazing exclusion on plant–soil C and N dynamics (Aynekulu et al., 2017; Shrestha & Stahl, 2008; Wu et al., 2017). Thus, there are no consistent effects of the enclosure on C and N storage at different grassland types, owing to the different soil texture, vegetation types, climate conditions, and historical grazing practices (Hu et al., 2016; Shang et al., 2017; Wu et al., 2017). In addition, there are few studies to show how habitat types affect the allocation of C and N storage in vegetation–soil system in different grassland ecosystems (Bi et al., 2018; Hu et al., 2016). To assess how habitat types affect the dynamic changes in C and N storage in vegetation and soil at degraded grassland is crucial to guide the reasonable and suitable measures for the ecosystem restoration and management.

Horqin Sandy Land was one of the most severe desertification areas in northern China before 1970. Mobile dune (MD), with more than 90% bare soil, dominated the desertified sandy grasslands. As the annual precipitation of 350–500 mm and abundant seed sources is remained in Horqin Sandy Land, grazing exclusions allow pioneer plants to establish and survive and then affect the grassland's succession. With the long‐term implement of grazing exclusion or grazing prohibition, most of MD gradually restored to the semi‐fixed (SFD) or fixed dunes (FD) (Guo et al., 2008; Li et al., 2013; Zuo et al., 2015). Many studies have shown that the restoration of degraded vegetation in sandy grassland ecosystems can increase plant biomass, the C and N storage of vegetation or soil (Chen et al., 2012; Li et al., 2012; Zuo et al., 2016). However, it is still unclear that how habitat types affect the allocation of C and N storage in vegetation and soil. Although the previous study has examined the changes in C and N storage in vegetation or soil at different sandy grassland habitats, there is still lack of the dynamic changes in allocation in C and N storage of vegetation–soil systems (Zuo et al., 2015). Therefore, to explore the effects of habitat types on the dynamic changes in allocation in C and N storage of vegetation–soil system in sandy grassland, ecosystems can enhance our understanding of how human activity affects the C and N cycling of sandy grassland.

In this study, we investigated a 5‐year dynamic changes in plant biomass, C and N concentrations, and storage of vegetation–soil system in three kinds of sandy grassland habitats. Specifically, we tested three hypotheses: (a) plant biomass, C and N storage of vegetation and soil increased from SFD to grasslands (G); (b) the allocation in the C and N storage of vegetation–soil system in three habitat type changes with sampling years; and (c) in different sandy grassland habitats, soil can potentially sequester considerable C and N.

## 2 MATERIALS AND METHODS

### 2.1 Study area

Our study was conducted in a semiarid sandy grassland in the south‐central part of Horqin Sandy Land (42°55′ N, 120°42′ E; 360 m elevation), Northern China. The site falls within a semiarid monsoon climate in the moderate temperature zone. The annual mean temperature and precipitation are, respectively, 7.1°C and 300 mm (2006–2020). More than eighty percent of the total precipitation occurs from June to August, and monthly mean temperature ranges from −12.6°C in January to 24.1°C in July. The annual effective accumulated temperature ≥10°C is more than 3,000°C. According to the different vegetation covers, the landscape is characterized by a mosaic of MD, SFD, FD, and G (Zuo et al., 2012). The soil is zonal and belongs to the chestnut soils in the Chinese classification or Orthi‐Sandic Entisols based on the FAO classification (Su et al., 2006). Wind blows at a velocity of 1.1 to 3.3 m s^−1^, with southwest to south and northwest directions prevailing. The effects of wind erosion on soil are very serious, which then strongly impacts vegetation distribution (Zuo et al., 2009). The dominant native plant species are *Corispermum macrocarpum* Bge. and *Artemisia halodendron* Turcz. ex Bess. in SFD with 10 ~ 60% vegetation cover, *Artemisia scoparia* Waldst. et Kit. and *Cleistogenes squarrosa* (Trin.) Keng in FD with more than 60% vegetation cover, and finally *A. scoparia* and *Pennisetum centrasiaticum* Tzvel. in G with more than 60% vegetation cover.

### 2.2 Experimental design and measurements

We selected 18 sampling sites corresponding to the SFD, FD, and G habitat types under the condition of long‐term enclosure, within 6 replication sites in each habitat, and the distance among all sites was about 0.5–8 km. A homogeneous 20 × 20 m plot was established at each site. Both SFD and FD were naturally restored from mobile dunes by erecting fenced enclosures with 2‐m‐high columns and barbed wire in 1995 and 1980, respectively. G was also excluded livestock by fencing since 1996, which was regarded as a better vegetation type than other grassland habitat types in our study area.

Five 1 × 1 m quadrats were established at the center and four corners of each plot at each site. On each quadrat, we conducted the plant and soil sampling in Mid‐August of 2011, 2013, and 2015. Within each quadrat, we collected the aboveground plant biomass of each species by the method of mowing and litter mass. Roots in four layers (0–10, 10–20, 20–40, and 40–60 cm) were also sampled by using a soil auger (10 cm‐diameter) in each quadrat. We used a soil auger equipped with a stainless‐steel cylinder and collected three samples in 5 cm increments (down to 60 cm) to determine the bulk densities of four layers (0–10, 10–20, 20–40, and 40–60 cm), and calculated the average of the three samples as a measure of bulk density in each site (Li et al., 2013).

We washed the roots to remove soil and other debris. We dried the aboveground biomass, litter mass, and root biomass at 60°C for 48 hr, and soil used for examining bulk density was dried at 105°C for 24 hr. The plant and soil samples were ground by a mill, and after that we measured the C and N concentrations in aboveground plant, litter, root, and soil by an elemental analyzer (Costech ECS 4010). We calculated aboveground plant, litter and root biomass, bulk density, C, and N concentrations as the mean from five quadrats in each plot. We converted % C and % N into g C m^−2^ and g N/m^2^ of C and N storage in aboveground plant, litter, and root by using the biomass on a per area unit basis, and transformed % C and % N into g C m^−2^ and g N/m^2^ of soil C and N storage by using soil bulk density and soil depth. We calculated the storage of C and N in the vegetation (aboveground plant, litter, and root)–soil system in each plot by averaging the data from five quadrats.

### 2.3 Data analysis

The two‐way analysis of variance (ANOVA) was used to examine the effects of habitat types, sampling years, and their interaction on aboveground biomass, litter mass, root biomass, soil bulk density, C and N concentrations, and storage in aboveground plant, litter, root, and soil at each depth, vegetation system, and vegetation–soil system. Least significant differences (LSD) test was used to compare the different habitat types or sampling years if the ANOVA was significant (*p* < .05). All statistical analyses were performed by SPSS (version 19.0).

## 3 RESULTS

### 3.1 Dynamic changes in plant biomass, C and N concentrations in vegetation system

Habitat types significantly affected plant biomass, C and N concentrations of aboveground plant and root, and C concentrations of litter (Table 1, *p* < .05). The mean aboveground plant biomass, litter mass, and total root biomass (0–60 cm) increased by 1.57, 2.85, and 1.72% from SFD to G, respectively (Table 2, *p* < .05). However, the 5‐year short‐term changes for plant biomass varied with the habitat types. The effects of sampling years were significant on litter mass, C and N concentrations in aboveground plant and litter, and root N concentrations (Table 1, *p* < .05). Aboveground plant biomass in SFD and G did not significantly change from 2011 to 2015, while litter mass in SFD significantly increased from 2011 to 2015 (Table 2, *p* < .05). The root biomass did not differ among SFD, FD, and G from 2011 to 2015 in the layers of 0–10, 10–20, and 20–40 cm (Table 3, *p* < .05). The total root biomass (0–60 cm) in SFD was higher in 2013 than in 2011 and 2015, while it was higher in 2015 than in 2011 and 2013 in FD (Table 3, *p* < .05). The interaction between habitat types and sampling years significantly affected aboveground plant biomass and its N concentrations (Table 1, *p* < .05).

**TABLE 1:** Effects of habitat types, sampling years, and their interaction on the plant biomass, C and N concentrations and storage in vegetation–soil ecosystem

	Habitat types	Sampling years	Habitat types * Sampling years
Aboveground biomass	11.86***	2.79	6.71***
Aboveground plant C concentration	9.70***	5.68**	2.58
Aboveground plant N concentration	11.95***	6.31**	6.56***
Aboveground plant C storage	10.16***	3.55*	8.09***
Aboveground plant N storage	13.35***	3.54*	4.99**
Litter mass	21.53***	5.56**	0.02
Litter C concentration	16.11***	5.56**	1.55
Litter N concentration	1.77	5.95**	0.19
Litter C storage	18.79***	4.79*	0.08
Litter N storage	22.06***	9.92***	0.01
Root biomass	3.25*	0.17	1.03
Root C concentration	8.04**	2.60	0.97
Root N concentration	23.89***	5.34**	2.54
Root C storage	2.46	0.24	1.05
Root N storage	5.29**	1.13	0.64
Soil bulk density	19.28***	10.43***	1.76
Soil C concentration	56.83***	5.74**	1.33
Soil N concentration	158.11***	32.89***	5.87**
Soil C storage	66.16***	4.59*	0.83
Soil N storage	108.96***	8.64**	2.21
Vegetation system C storage	14.68***	0.55	2.80*
Vegetation system N storage	28.07***	6.17**	2.92*
Vegetation–soil system C storage	67.63***	4.15*	0.68
Vegetation–soil system N storage	112.96***	9.09**	2.08

Note

Different asterisk indicates the significant differences in the effects of habitat types, sampling years, and their interaction on plant biomass, C and N concentrations and storage in vegetation–soil ecosystem.

* *p* < .05

** *p* < .01

*** *p* < .001.

**TABLE 2:** Changes in aboveground biomass, litter biomass, and their carbon (C) and nitrogen (N) concentrations in three habitat types among different years (Means ± *SE*, *n* = 6)

	2011			2013			2015		
	SFD	FD	G	SFD	FD	G	SFD	FD	G
Aboveground plant									
Biomass (g m^−2^)	87.95 ± 3.01^cA^	264.66 ± 12.80^aA^	179.45 ± 18.47^bA^	98.10 ± 7.27^bA^	131.41 ± 8.20^bB^	190.38 ± 19.15^aA^	160.16 ± 32.87^aA^	149.98 ± 8.21^aB^	175.04 ± 16.48^aA^
C concentration (g kg^−1^)	433.77 ± 5.91^aA^	408.53 ± 7.09^bA^	431.43 ± 2.77^aA^	418.13 ± 1.75^bA^	407.59 ± 2.52^cA^	424.33 ± 0.82^aA^	422.29 ± 7.31^aA^	403.27 ± 7.07^bA^	403.10 ± 4.02^bB^
N concentration (g kg^−1^)	11.67 ± 0.38^bB^	14.33 ± 1.02^bB^	18.63 ± 1.36^aA^	15.56 ± 0.19^aA^	16.09 ± 0.041^aB^	16.61 ± 0.39^aA^	15.65 ± 1.35^bA^	19.44 ± 0.60^aA^	16.67 ± 0.47b^bA^
Litter									
mass (g m^−2^)	23.29 ± 4.19^cB^	42.65 ± 0.57^bA^	132.45 ± 7.59^aA^	63.75 ± 9.66^bA^	71.88 ± 15.93^bA^	166.34 ± 12.07^aA^	87.34 ± 9.78^bA^	100.19 ± 26.49^bA^	198.04 ± 35.72^aA^
C concentration (g kg^−1^)	436.03 ± 3.95^aAB^	372.60 ± 14.55^bA^	397.57 ± 12.33^abA^	463.22 ± 3.81^aA^	400.02 ± 11.43^bA^	409.65 ± 8.37^bA^	415.38 ± 14.08^aB^	349.68 ± 22.31^bA^	411.39 ± 6.81^aA^
N concentration (g kg^−1^)	8.93 ± 0.61^aB^	11.23 ± 1.19^aAB^	10.67 ± 0.34^aA^	7.83 ± 0.18^bB^	9.85 ± 0.75^aB^	10.64 ± 0.40^aA^	12.01 ± 0.42^aA^	13.27 ± 0.70^aA^	12.90 ± 2.91^aA^

Note

Different lowercase letters indicate the significant difference in same variable among different habitat types in the same year at *p* < .05. Different capital letters indicate the significant difference in same variable among different years in the same habitat type at *p* < .05.

Abbreviations: FD, fixed dunes; G, grasslands; SFD, semi‐fixed dunes.

**TABLE 3:** Changes in root biomass, root carbon (C), and nitrogen (N) concentrations in three habitat types among different years (Means ± *SE*, *n* = 6)

	2011			2013			2015		
	SFD	FD	G	SFD	FD	G	SFD	FD	G
0–10 cm									
Biomass (g m^−2^)	54.66 ± 31.26^aA^	129.04 ± 59.29^aA^	140.10 ± 72.24^aA^	112.98 ± 25.74^aA^	62.67 ± 18.91^aA^	122.52 ± 28.97^aA^	85.01 ± 39.44^aA^	129.67 ± 25.40^aA^	119.69 ± 31.70^aA^
C concentration (g kg^−1^)	434.30 ± 4.78^aB^	431.83 ± 5.55^aA^	391.80 ± 32.75^aA^	452.67 ± 2.40^aA^	431.30 ± 3.00^bA^	429.54 ± 3.40^bA^	446.01 ± 1.53^aA^	421.25 ± 8.00^abA^	415.55 ± 14.13^bA^
N concentration (g kg^−1^)	7.23 ± 0.03^bB^	11.07 ± 1.28^aA^	10.83 ± 0.97^aAB^	7.77 ± 0.16^cB^	11.89 ± 0.34^aA^	9.67 ± 0.52^bB^	9.71 ± 0.59^bA^	11.41 ± 0.65^abA^	11.98 ± 0.54^aA^
10–20 cm									
Biomass (g m^−2^)	13.84 ± 6.83^aA^	15.72 ± 2.89^aA^	35.53 ± 14.01^aA^	19.43 ± 5.43^bA^	13.64 ± 1.74^bA^	49.77 ± 9.66^aA^	13.39 ± 3.12^bA^	27.18 ± 9.95^abA^	37.42 ± 8.54^aA^
C concentration (g kg^−1^)	390.87 ± 51.66^aA^	455.00 ± 6.42^aA^	424.24 ± 5.14^aAB^	445.11 ± 7.25^aA^	440.90 ± 6.07^aAB^	434.23 ± 2.73^aA^	424.24 ± 8.85^aA^	401.71 ± 19.41^aB^	413.81 ± 8.75^aB^
N concentration (g kg^−1^)	7.13 ± 0.61^bB^	9.37 ± 0.58^abA^	10.56 ± 0.89^aA^	7.83 ± 0.42^bB^	10.94 ± 1.06^aA^	8.01 ± 0.67^bB^	11.01 ± 1.27^aA^	9.42 ± 0.29^aA^	11.88 ± 0.64^aA^
20–40 cm									
Biomass (g m^−2^)	17.37 ± 7.94^aA^	11.90 ± 1.87^aA^	20.59 ± 12.53^aA^	18.93 ± 5.52^bA^	15.72 ± 2.50^bA^	36.14 ± 7.16^aA^	12.02 ± 3.48^aA^	21.82 ± 3.80^aA^	21.92 ± 5.56^aA^
C concentration (g kg^−1^)	445.73 ± 3.27^aA^	431.67 ± 9.91^aA^	422.63 ± 9.02^aA^	439.93 ± 7.53^aA^	440.71 ± 6.14^aA^	430.07 ± 4.61^aA^	420.56 ± 5.10^aB^	403.38 ± 21.01^aA^	414.73 ± 8.44^aA^
N concentration (g kg^−1^)	7.03 ± 0.15^bB^	12.30 ± 0.53^aA^	10.77 ± 1.16^aAB^	10.33 ± 0.87^aAB^	11.16 ± 1.26^aA^	8.92 ± 0.73^aB^	12.01 ± 1.38^aA^	9.91 ± 0.27^aA^	11.94 ± 0.62^aA^
40–60 cm									
Biomass (g m^−2^)	4.44 ± 1.75^aB^	2.50 ± 0.65^aB^	3.18 ± 2.01^aA^	12.37 ± 2.40^aA^	13.82 ± 2.98^aA^	15.46 ± 4.12^aA^	3.15 ± 0.67^bB^	13.80 ± 1.14^abA^	29.83 ± 13.01^aA^
C concentration (g kg^−1^)	440.50 ± 4.39^aA^	436.27 ± 20.31^aA^	424.80 ± 34.71^aA^	432.51 ± 13.54^aA^	425.40 ± 8.91^aA^	439.63 ± 4.13^aA^	436.21 ± 7.22^aA^	386.75 ± 14.53^bB^	416.99 ± 6.20^abA^
N concentration (g kg^−1^)	7.45 ± 0.61^aB^	10.87 ± 2.05^aA^	10.50 ± 0.94^aA^	13.23 ± 1.77^aA^	11.57 ± 1.11^aA^	7.44 ± 0.39^bB^	11.49 ± 1.03^aAB^	11.77 ± 0.52^aA^	12.57 ± 0.64^aA^

Note

Different superscript lowercase letters indicate the significant difference in same variable among different habitat types in the same year at *p* < .05. Different superscript capital letters indicate the significant difference in same variable among different years in the same habitat type at *p* < .05.

Abbreviations: FD, fixed dunes; G, grasslands; SFD, semi‐fixed dunes.

The mean C concentration in aboveground plant and litter was lower in FD than that in other two habitats, and the mean N concentration in aboveground plant was lower in SFD than that in other two habitats, while the mean root C concentration (0–60 cm) was higher in SFD than that in other two habitats (Tables 2 and 3, *p* < .05). The short‐term changes in C and N concentrations in aboveground plant, litter, and root (0–60 cm) varied with the habitat types. The aboveground plant C concentration in G was lower in 2015, and the aboveground plant N concentrations in SFD and FD, as well as litter N concentration in SFD, significantly increased from 2011 to 2015 (Table 2, *p* < .05). The root C concentration in SFD increased from 2011 to 2015 in the layer of 0–10 cm, while it showed a reversed trend in the layer of 20–40 cm. The root C concentrations in FD decreased from 2011 to 2015 in the layers of 10–20 and 40–60 cm. The root N concentrations in SFD significantly increased from 2011 to 2015 in the layers of 0–10, 10–20, and 20–40 cm, while root N concentration at each layer was not differed among sampling years in FD and it was much lower in 2013 in G (Table 2).

### 3.2 Dynamic changes in soil bulk density, C and N concentrations in soil

Habitat types and sampling years significantly affected soil bulk density, soil C and N concentrations, while their interaction only significantly affected soil N concentration (Table 1, *p* < .01). The soil bulk densities (layers of 0–10 cm, 20–40 cm, and 40–60 cm) in 2013 and 2015 significantly decreased from SFD to G, as well as the soil bulk density in the layer of 40–60 cm in 2011 (Table 4, *p* < .05). The soil C and N concentration in each layer (0–10 cm, 10–20 cm, 20–40 cm, and 40–60 cm) significantly increased from SFD to G. In addition, the 5‐year's short‐term changes in soil bulk density, soil C and N concentration varied with the habitat types. The soil bulk density of SFD in each layer, soil bulk density of FD in the layer of 0–10 and 10–20 cm, and soil bulk density of G at 0–10 cm significantly decreased from 2011 to 2015. There were no significantly changes in soil C concentrations in SFD and FD at each layer in a 5 year's short‐term period (Table 4, *p* > .05), while the topsoil N concentration (layer of 0–10 cm) in SFD and soil N concentrations (layers of 0–10 and 10–20 cm) in FD and G significantly increased from 2011 to 2015 (Table 4, *p* < .05).

**TABLE 4:** Changes in soil bulk density, carbon (C), and nitrogen (N) concentrations in three habitat types among different years (Means ± *SE*, *n* = 6)

	2011			2013			2015		
	SFD	FD	G	SFD	FD	G	SFD	FD	G
0–10 cm									
Bulk density (g cm^−3^)	1.61 ± 0.01^aA^	1.59 ± 0.03^aA^	1.54 ± 0.04^aA^	1.57 ± 0.01^aAB^	1.50 ± 0.02^aB^	1.33 ± 0.04^bB^	1.53 ± 0.01^aB^	1.49 ± 0.02^aB^	1.36 ± 0.04^bB^
C concentration (g kg^−1^)	0.77 ± 0.15^bA^	3.07 ± 0.26^aA^	2.97 ± 0.12^aB^	1.10 ± 0.08^cA^	3.94 ± 0.19^bA^	5.17 ± 0.29^aA^	0.85 ± 0.13^bA^	3.41 ± 0.58^aA^	4.33 ± 0.58^aAB^
N concentration (g kg^−1^)	0.16 ± 0.01^bB^	0.39 ± 0.01^aB^	0.40 ± 0.02^aC^	0.15 ± 0.01^cB^	0.47 ± 0.02^bB^	0.61 ± 0.03^aB^	0.21 ± 0.01^bA^	0.63 ± 0.04^aA^	0.71 ± 0.03^aA^
10–20 cm									
Bulk density (g cm^−3^)	1.59 ± 0.01^aA^	1.59 ± 0.01^aA^	1.55 ± 0.05^aAB^	1.61 ± 0.01^aA^	1.64 ± 0.02^aA^	1.60 ± 0.02^aA^	1.53 ± 0.02^aB^	1.49 ± 0.03^aB^	1.46 ± 0.04^aB^
C concentration (g kg^−1^)	0.62 ± 0.07^bA^	1.55 ± 0.12^aA^	1.79 ± 0.23^aB^	0.80 ± 0.05^cA^	2.11 ± 0.18^bA^	2.97 ± 0.38^aAB^	0.73 ± 0.09^cA^	1.76 ± 0.18^bA^	3.69 ± 0.50^aA^
N concentration (g kg^−1^)	0.15 ± 0.01^bA^	0.24 ± 0.02^aB^	0.29 ± 0.04^aB^	0.14 ± 0.01^bA^	0.29 ± 0.02^aB^	0.35 ± 0.04^aB^	0.16 ± 0.01^cA^	0.44 ± 0.01^bA^	0.54 ± 0.03^aA^
20–40 cm									
Bulk density (g cm^−3^)	1.59 ± 0.01^aA^	1.59 ± 0.02^aAB^	1.53 ± 0.05^aA^	1.61 ± 0.01^abA^	1.63 ± 0.01^aA^	1.56 ± 0.04^bA^	1.55 ± 0.02^aB^	1.55 ± 0.03^aB^	1.41 ± 0.06^bA^
C concentration (g kg^−1^)	0.60 ± 0.11^bA^	1.14 ± 0.14^bA^	2.49 ± 0.56^aA^	0.63 ± 0.03^bA^	1.63 ± 0.18^bA^	3.19 ± 0.64^aA^	0.58 ± 0.08^bA^	1.34 ± 0.22^bA^	2.92 ± 0.50^aA^
N concentration (g kg^−1^)	0.14 ± 0.02^bA^	0.20 ± 0.02^bB^	0.39 ± 0.07^aA^	0.12 ± 0.01^bA^	0.22 ± 0.02^bB^	0.36 ± 0.05^aA^	0.13 ± 0.01^cA^	0.33 ± 0.01^bA^	0.41 ± 0.03^aA^
40–60 cm									
Bulk density (g cm^−3^)	1.58 ± 0.02^aAB^	1.53 ± 0.02^abB^	1.47 ± 0.02^bA^	1.59 ± 0.01^aA^	1.62 ± 0.01^aA^	1.47 ± 0.06^bA^	1.54 ± 0.02^aB^	1.52 ± 0.01^aB^	1.36 ± 0.05^bA^
C concentration (g kg^−1^)	0.61 ± 0.09^bA^	0.88 ± 0.17^abA^	2.80 ± 0.95^aA^	0.56 ± 0.03^bA^	1.54 ± 0.19^bA^	4.23 ± 1.27^aA^	0.49 ± 0.07^cA^	1.11 ± 0.19^bA^	2.29 ± 0.27^aA^
N concentration (g kg^−1^)	0.14 ± 0.01^bA^	0.19 ± 0.01^bA^	0.40 ± 0.10^aA^	0.11 ± 0.01^bA^	0.23 ± 0.02^bA^	0.42 ± 0.09^aA^	0.12 ± 0.01^cA^	0.22 ± 0.01^bA^	0.34 ± 0.02^aA^

Note

Different superscript lowercase letters indicate the significant difference in same variable among different habitat types in the same year at *p* < .05. Different superscript capital letters indicate the significant difference in same variable among different years in the same habitat type at *p* < .05.

Abbreviations: FD, fixed dunes; G, grasslands; SFD, semi‐fixed dunes.

### 3.3 Dynamic changes in C and N storage in vegetation–soil system

**FIGURE 1:**
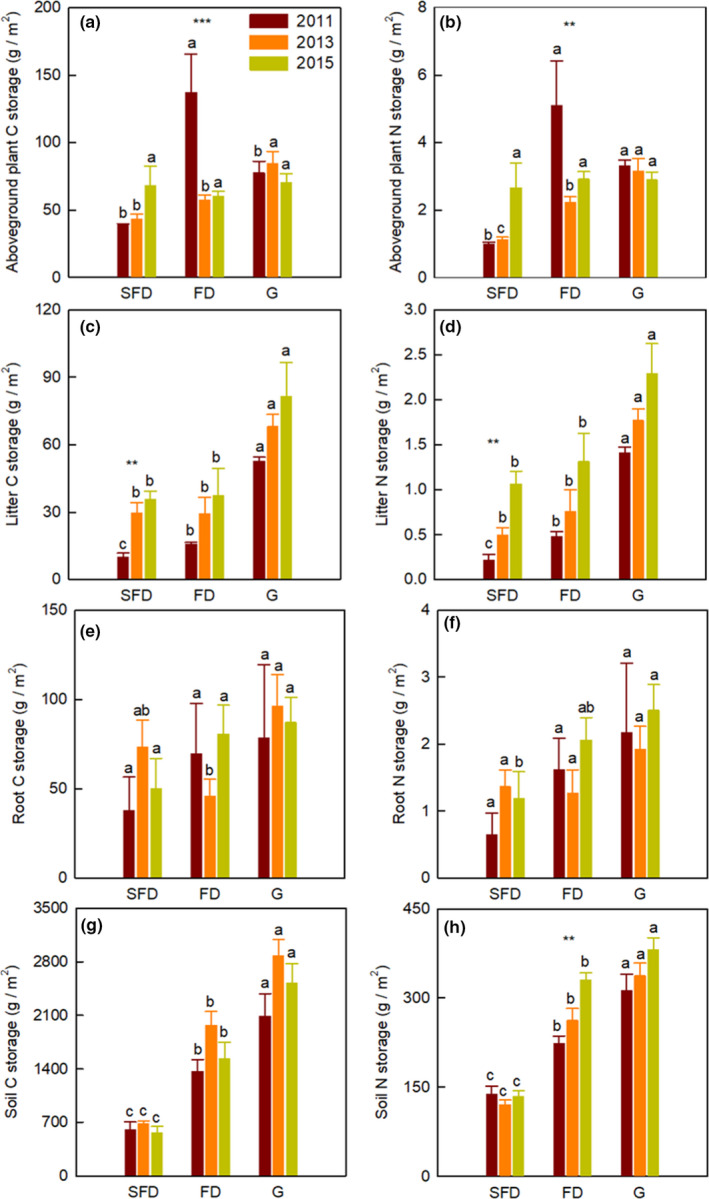
The allocation of C and N storage in vegetation–soil system among three habitat types. Different lowercase letters indicate the significant difference in same year among different habitat types. The significant differences in same habitat type among different years are indicated by asterisk, **p < .01, ***p < .001. SFD, semi‐fixed dunes; FD, fixed dunes; G, grasslands

**FIGURE 2:**
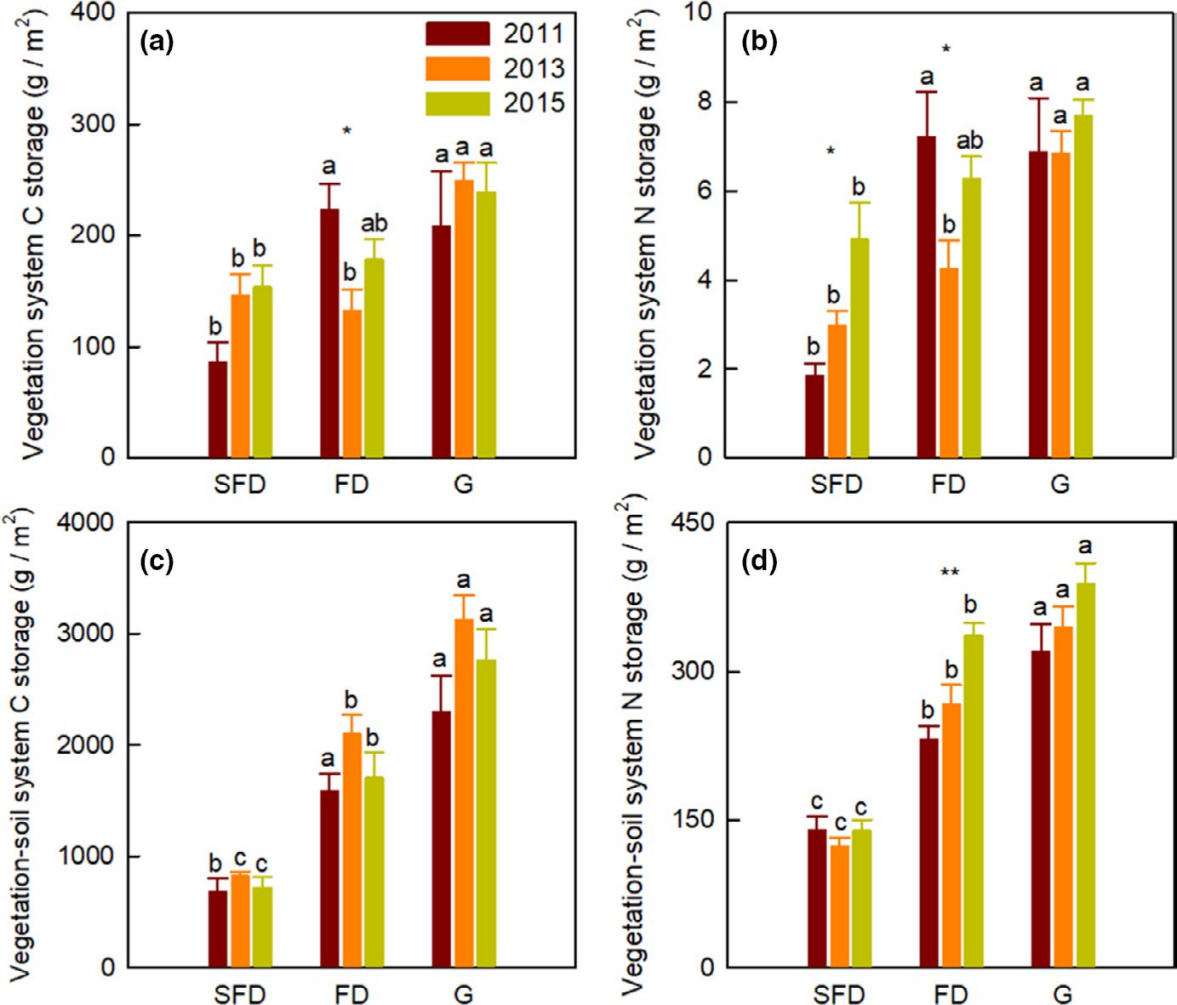
Changes in C and N storage in vegetation system and vegetation–soil system among three habitat types. Different lowercase letters indicate the significant difference in same year among different habitat types. The significant differences in same habitat type among different years are indicated by asterisk, *p < .05, **p < .01. SFD, semi‐fixed dunes; FD, fixed dunes; G, grasslands

**FIGURE 3:**
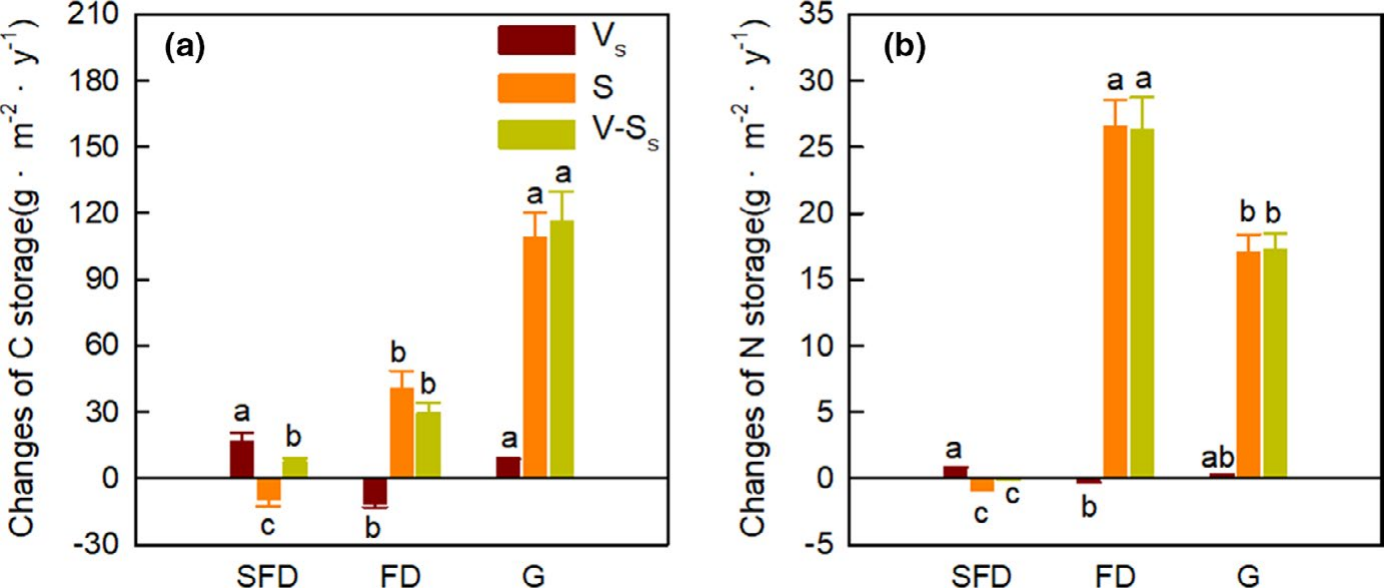
The annual increase rates of C and N storage in vegetation–soil system from 2011 to 2015 among three habitat types. Different lowercase letters indicate the significant difference in same variable among different habitat types. V_s_, vegetation system; S, soil; V‐S_s_, vegetation–soil system. SFD, semi‐fixed dunes; FD, fixed dunes; G, grasslands

Habitat types and sampling years significantly affected the C and N storage in aboveground plant, litter, soil, and vegetation–soil system, while their interaction showed an obvious influence only on C and N storage in aboveground plant and vegetation system (Table 1; Figures 1 and 2, *p* <.05). The C and N storage in aboveground plant, litter, root and soil (0–60 cm), vegetation system, and vegetation–soil system significantly increased from SFD to G (Figures 1a–h and 2a–d, *p* < .05). The C and N storage of aboveground plant in FD was higher in 2011 than in 2013 and 2015 (Figure 1a,b, *p* < .001). The litter C and N storage and vegetation system N storage in SFD remarkably increased from 2011 to 2015 (Figures 1c,d and 2b, *p* < .05). The root C and N storage did not differ in each habitat among 2011, 2013, and 2015 (Figure 1e,f, *p* > .05). In addition, the N storage of soil and vegetation–soil system in FD increased significantly from 2011 to 2015, as well as the N storage of vegetation–soil system in SFD (Figures 1h and 2d, *p* < .05). The annual increasing rate of C storage in soil and vegetation–soil system in G was significantly higher than the other two habitat types (Figure 3a, *p* < .05). Additionally, the annual increasing rate of vegetation system C and N storage in SFD was higher than FD, while the annual increasing rate of N storage in soil and vegetation–soil system in FD was significantly higher than the other two habitat types (Figure 3).

## 4 DISCUSSION

### 4.1 Vegetation restoration enhances plant biomass and alters the C and N concentrations of vegetation system

Our findings illustrated that the mean plant biomass (aboveground plant, litter, and root) consistently increased from SFD to G, and the result was in agreement with previous study which demonstrated that plant, litter biomass increased with advancing sandy grassland restoration in semiarid sandy grassland (Zuo et al., 2015, 2017). However, the changing trend of aboveground plant biomass from SFD to G was not consistent in each year, possibly because the annual herbs are dominated in sandy grassland and sensitive to precipitation changes (Sun et al., 2019; Zuo et al., 2012). The lower rate of litter decomposition in desertified sandy grassland ecosystem promotes the accumulation of litter mass from 2011 to 2015 (Li et al., 2016). In addition, no significant difference was found in root biomass either in habitat type or in sampling year at the four layers, and the result was similar to the previous study, which demonstrated that the root biomass is very stable and not sensitive to environmental changes (Fortier et al., 2013). Moreover, we found the aboveground plant biomass in SFD was lower than that in the other two habitats, whereas the root biomass did not significantly change among habitat types, which possibly because that the plants in SFD had to allocated more biomass to the belowground to absorb the limited soil water then maintaining their growth and development due to the low soil water content in SFD (Zuo et al., 2015).

Our study found that the C concentrations in aboveground plant, litter, and root (0–60 cm) reduced from SFD to G, while their N concentrations increased from SFD to G mainly due to the shrub of *Artemisia halodendrom* growing only in SFD, which has the higher C concentration but lower N concentration. In addition, we also found that the short‐term changes in C and N concentrations in vegetation system depended on habitat types; concomitantly, there are higher C concentration and relatively lower N concentration of aboveground plant, litter, and root in SFD, mainly due to different plant community composition with habitat restoration in semiarid sandy grassland. Plant community composition changes (from *Artemisia halodendrom* dominated in SFD eventually to annual and perennial herbs dominated in FD and G) will be able to alter plant photosynthesis and soil nutrient utilization, which consequently cause the variations in nutrient allocation of plant community (Hbirkou et al., 2011; Houghton, 1995; Zhang et al., 2018; Zuo et al., 2015). The plants in three habitats (such as *Artemisia halodendrom*) have formed certain characteristics and chemical composition to adapt to the environmental condition changes (He et al., 2015). The fragile environments such as infertile soil, semiarid climate, and strong wind erosion in this region also contribute to the changes in C and N concentrations in vegetation system in different sandy grassland habitats (Li et al., 2019).

### 4.2 Vegetation restoration decreases soil bulk density and increases soil C and N concentrations

The soil bulk density significantly decreased from SFD to G. Enclosure of sandy grassland eliminates the livestock's trample and decreases soil compaction that closely related to soil bulk density (Oduor et al., 2018; Su et al., 2005), consequently increasing the soil porosity and infiltration and finally leading to the accumulation of C and N in soil (Bach et al., 2012). The restoration of degraded vegetation enhances the above/belowground biomass and the accumulation of litter mass, further affecting the inputs of soil organic matters which plays a crucial role in the soil structure (Bach et al., 2012; Ren et al., 2018). Our study showed that more than 60% of root biomass was allocated in the layer of 0–10 cm, which also caused the decrease in soil bulk density in sandy grassland (Su et al., 2005). We also found that the 5‐year short‐term changes in soil bulk density depend on habitat type changes. The restoration of degraded vegetation decreased the soil bulk density of SFD (the mid‐term stage of dune fixation) in each layer, while the restoration of degraded vegetation only affects the soil bulk density of FD (the late‐term stage of dune fixation) and G in the 0–10 cm of the surface soil.

Our study also displayed that the soil (depth in 0–60 cm) C and N concentrations increased significantly from SFD to G, the soil organic matter concentration increased with vegetation restoration, which consequently amplified the soil C and N concentrations in semiarid sandy grassland. The result is in general agreement with previous studies, which demonstrated that the C and N concentrations positively related to the plant biomass (Fornara & Tilman, 2008; Zuo et al., 2015). We also found that soil C concentrations in three habitats did not significantly change from 2011 to 2015, while the N concentration of SFD in the layer of 0–10 cm and that of FD and G in the layer of 0–20 cm significantly increased from 2011 to 2015. These results suggest that soil C concentrations in sandy grassland are relative stable in a short‐term period, while continuous restoration of vegetation enhances the N accumulation in soil, even though the improvement of soil is only limited in the shallow soil (Wang et al., 2020; Zuo et al., 2018).

### 4.3 Habitat types significantly affected the dynamic changes in C and N storage allocation of vegetation–soil system

Our findings illustrated that the C and N storage of aboveground plant, litter, root (depth in 0–60 cm), soil (depth in 0–60 cm), vegetation system, and vegetation–soil system increased gradually from SFD to G (Zuo et al., 2015). The plant biomass increases amplified the plant C and N storage with vegetation restoration, and the input of soil organic matter increases stimulated the soil C and N concentrations, consequently increasing the soil C and N storage from SFD to G with vegetation restoration. Consistent with the changing trend of aboveground biomass and vegetation system biomass, we also found that the C and N storage of aboveground plant and vegetation system fluctuated with years, while the litter C and N storage increased from 2011 to 2015. These results demonstrate the hypothesis that C and N sequestration potential of ecosystem is highly correlated with C and N biomass production of vegetation system (De Deyn et al., 2009; Wu et al., 2008). Changes in plant community composition of vegetation system also enhance the C and N storage in vegetation system with the restoration of degraded sandy grassland (Zuo et al., 2015).

Larger plant biomass enhances aboveground litter and belowground root inputs, with consequently effects on the C and N sequestration potential of soil under litter decomposition (Knops et al., 2007). Herbaceous litter and dead roots play an important role in soil C and N storage (Wolkovich et al., 2010). Our result illustrated that the N storage of soil and vegetation–soil system apparently increased in a 5 year's short‐term period due to the accumulation of litter mass in 5 years. In semiarid sandy grassland, more than 81.90%–98.03% of C and N were sequestered in soil, suggesting that soil has the considerable C and N sequestration potential in the restoration of degraded sandy grassland (Guo et al., 2008; Sartori et al., 2007; Wu et al., 2010). The C and N storage in vegetation system of SFD and the soil N storage in FD increased from 2011 to 2015, suggesting that more C and N are sequestered in vegetation system in the mid‐stage of dune fixation, while more N is sequestered in soil in the late‐stage of dune fixation. The main reason for the annual increasing rate of soil C storage in G was higher than other two habitat types was soil texture, especially the proportion of silt and clay (Frasier et al., 2019). As the last stage of dune fixation, FD has the higher species richness and the existence of sand‐fixation Leguminosae shrub (*Caragana microphylla* Lam.) may cause the higher annual increasing rate of soil N storage (Brantley & Young, 2010; Chen & Stark, 2000).

## 5 CONCLUSIONS

We found that the C and N storage of vegetation system (aboveground plant, litter, root), soil, and vegetation–soil system increased from SFD to G, while the 5‐year short‐term changes in C and N storage in vegetation–soil system varied with the habitat types. In the restoration of degraded sandy grassland, the N storage of vegetation system in SFD, as well as the N storage of soil and vegetation–soil system in FD, increased from 2011 to 2015, while the C storage in vegetation–soil system did not change in a short period of 5 years with vegetation restoration. Similar to the C storage changes in dune habitats, the C and N storage in grassland habitat kept the relative stability in 5 years. These results highlight the crucial differences in C and N storage of vegetation–soil system responding to habitat variation. We also found that the most of C and N were stored in soil at three different habitats, suggesting that the soil has the larger C and N sequestration potential in sandy grassland ecosystems. To our knowledge, this study has illustrated that how habitat type variations affect the dynamic changes in allocation in C and N storage of vegetation–soil system in a sandy grassland, which has an important application for degraded vegetation restoration and management in sandy grassland ecosystems. To exclude livestock grazing or implement, long‐term enclosure can promote the degraded vegetation restoration and its N storage in semi‐fixed dune, as well as N storage of soil and vegetation–soil system in fixed dune.

## CONFLICT OF INTEREST

None declared.

## AUTHOR CONTRIBUTION

**Peng Lv:** Conceptualization (equal); Data curation (equal); Formal analysis (lead); Investigation (lead); Methodology (equal); Writing‐original draft (lead); Writing‐review & editing (equal). **Shanshan Sun:** Data curation (supporting); Investigation (supporting). **Eduardo Medina‐Roldánd:** Conceptualization (supporting); Writing‐original draft (supporting); Writing‐review & editing (supporting). **Shenglong Zhao:** Data curation (supporting); Investigation (supporting). **Ya Hu:** Data curation (supporting); Investigation (supporting). **Aixia Guo:** Data curation (supporting); Investigation (supporting). **Xiaoan Zuo:** Conceptualization (equal); Data curation (equal); Formal analysis (equal); Investigation (equal); Writing‐original draft (equal); Writing‐review & editing (equal).

## Data Availability

I have archived my data to Dryad and completed the data accessibility statement, accessible at https://doi.org/10.5061/dryad.9w0vt4bfs.
